# Urine diversion dry toilets in eThekwini Municipality, South Africa: acceptance, use and maintenance through users’ eyes

**DOI:** 10.2166/washdev.2017.079

**Published:** 2017-01-02

**Authors:** Nosipho Mkhize, Myra Taylor, Kai M. Udert, Teddy G. Gounden, Chris A. Buckley

**Affiliations:** 1 **Nosipho Mkhize** (corresponding author), **Teddy G. Gounden** eThekwini Water and Sanitation, 3 Prior Road, Durban 4000, South Africa; 2 **Nosipho Mkhize**, **Myra Taylor** Discipline of Public Health Medicine, School of Nursing and Public Health, University of KwaZulu-Natal, Durban 4041, South Africa; 3 **Kai M. Udert** Eawag, Swiss Federal Institute of Aquatic Science and Technology, Überlandstrasse 133, Dübendorf 8600, Switzerland; 4 **Chris A. Buckley** Pollution Research Group, School of Engineering, University of KwaZulu-Natal, Durban 4041, South Africa

**Keywords:** education, focus group discussions, qualitative, quantitative, re-use, sanitation

## Abstract

This study was part of the VUNA project aimed to develop an affordable sanitation system that produces a valuable fertiliser, reduces pollution of water resources and promotes health. Urine diversion dry toilets (UDDTs) simplify the on-site hygienisation of faeces and allow for nutrient recovery from urine. Social acceptance is vital for the implementation of the UDDT, because sanitation is only effective if the system not only provides a well-designed toilet and effective waste management, but also offers users a facility that caters to their needs and is sensitive to their cultural lifestyle. This study used qualitative and quantitative methods to investigate acceptance, use and maintenance of UDDTs. Key findings indicate lower levels of acceptance of UDDTs among the elderly, who are accustomed to traditional pit toilets. The users aspire to own a flush toilet, perceived to be indicative of household wealth. A dominant concern was emptying the pit and the quality of the building material. Community interventions are required that will promote acceptance, understanding and encourage proper use and maintenance of the UDDT, and may need some technology modification. There is an urgent need for increased community participation to address users’ perceptions, attitudes and behaviour concerning the UDDT.

## INTRODUCTION

The VUNA project is a collaboration between the Swiss Federal Institute of Aquatic Science and Technology (Eawag), eThekwini Water and Sanitation (EWS), the University of KwaZulu-Natal (UKZN), and the Swiss Federal Institutes of Technology in Zurich (ETHZ) and Lausanne (EPFL) (Etter et al. 2015). The project was multi-disciplinary in nature, and this study was part of the social acceptance component aiming to promote acceptance, proper use and maintenance of the urine diversion dry toilet (UDDT). The project is vital for South Africa because it is a water-scarce country, and this compels the country to initiate and develop adaptive mechanisms for water conservation and wastewater management.

Lack of clean water and basic sanitation is a challenge to service delivery and to poverty alleviation and sustainable development. In South Africa, 15 million people, most of whom live in rural areas, have no access to basic sanitation. A census of the South African government in 2011 reported that approximately 1.3 million households in South Africa are without access to piped water, of which the majority are black households (Stats SA 2011). Moreover, approximately 749,000 households in the country have no toilet system at all, while approximately 8,240,000 have flush toilets connected to a sewage system (Stats SA 2011). Consequently, both have serious health impacts. Baker & Ensink (2012) reported that access to sanitation can significantly reduce morbidity from helminth infections as 1.8 million people die every year from these diseases, the vast majority of whom are children under five. The Department of Water and Forestry DWAF (2006) advised that sanitation technology needs to be carefully chosen based on the permanence of the settlement, the technical aspects, financial costs, design, expectations and the environmental considerations. DWAF (2006) goes on to state that sanitation systems should protect the environment and not harm it.

One of the country’s Millennium Development Goals (MDGs) aimed to halve the number of people who are with-out water and sanitation (SAMDG 2011). From scientific predictions, by 2050 South Africa will experience a progressive decrease of economically usable freshwater resources; the country thus requires innovative sanitation technologies that are sensitive to the reality of water scarcity (Roma *et al*. 2013). Drangert *et al*. (2002) emphasised that alternative waste management options are needed to reconceptualise sanitation, from the ‘drop-flush-forget’ model to the protection of environmental pollution at source by means of ‘drop and re-use’ models. In South Africa, few studies have been undertaken regarding the acceptance, use and maintenance of such systems as the UDDT. This study follows on the earlier studies undertaken in the rural areas of eThekwini when the UDDTs were first installed, which contributed towards the design modifications over the years (Kvalsvig & Ngcoya 2006).

## PROVISION OF UDDT BY ETHEKWINI MUNICIPALITY

To respond to the urgent need, the eThekwini Municipality (EM) in 2002 installed UDDTs in its rural areas (Roma *et al*. 2011). Gounden *et al*. (2006) explains that the decision to opt for the UDDT was due to cost consideration and environ-mental impact compared to the flush toilet that people aspire towards, open defecation (OD), and the ventilated improved pit (VIP) that the community was using, which was harmful to the environment. The UDDTs provide the following benefits: (1) waterless operation; (2) no odour when correctly used and maintained; (3) treated faecal matter is dry, odourless and less offensive; (4) does not attract flies or other vectors; (5) treated faecal matter is partially sanitised and safer to handle; (6) aboveground design or use of containers in belowground vaults makes emptying simple; (7) minimal risk of contamination of ground and sur-face water resources; (8) possibility of aboveground design facilitates construction in challenging environments; and (9) possibility of construction in close proximity to or inside of the home adds security and convenience for users (Rieck *et al*. 2012).

The UDDT installation aimed to address the expansion of municipal boundaries from 1,366 to 2,297 km^2^, encapsulating a population of 3.5 million. The new areas were rural or periurban comprising approximately 75,000 houses, 80% of which had no appropriate water or sanitation facilities (Gounden *et al*. 2006). EM is in KwaZulu Natal, where there was a cholera outbreak between August 2000 and July 2001 with 105,389 registered cases and 219 documented deaths (Mudzanani et al. 2003). There was thus an urgency to implement the integrated water and sanitation project followed by health and hygiene education and training (Roma *et al*. 2011). The South African constitution preserves a basic right to receive sufficient water, stating that ‘… *Everyone has the right to have access to sufficient food and water* …’ (SA Constitution 1996).

The EM provides UDDTs as permanent assets of house-holds and communities. However, the sustainability of the UDDTs is dependent on the users’ acceptance, use and maintenance. Jackson (2005) states that such systems are to minimise environmental and health risks related to inadequate and poor sanitation. The UDD toilet is a sealed unit, so the groundwater is not impacted (Gounden et al. 2006). UDDT technology is based on the assumption that keeping urine and faeces separate destroys the diseasecausing pathogens contained in the faecal matter over time, through a drying process (Tilley *et al*. 2014). From the municipal perspective, one reason for choosing the UDDT rather than VIPs is that VIPs demand mechanical desludging, which requires expensive equipment that is vulnerable to failure, often cannot access the site, and frequently cannot cope with the heavy sludge and solid matter found in the pit (WIN-SA 2006).

## INVOLVING PEOPLE IN THE DELIVERY OF SANITATION

The sanitation policy of South Africa emphasises that sanitation is more than just the provision of toilets, but is a cohesive approach that embraces organisational, technical, financial, environmental, social and educational frameworks (DWAF 1996). McConville & Rosemarin (2012) emphasised that the latter can only be realised if people are part of proper planning and are involved in choosing the sanitation technology. It is only then that sanitation sustainability can be realised.

Providing people with toilets is insufficient, since measures also need to be taken to ensure that people accept, understand and properly use and maintain the toilet. Therefore, EM engaged in various steps to introduce the UDDT to the targeted communities; first, buy-in was sought from the ward councillors (local leadership), and the local leadership then introduced this to their respective communities (Kvalsvig & Ngcoya 2006). Moreover, health and hygiene education was provided before and after the installation of the toilet to systematically introduce the UDDTs as the new technology in communities, to ensure that the community accept and use the toilet properly. How-ever, Austin *et al*. (2005) state that education methods should be inclusive in nature, and take socio-cultural aspects into account. According to Parker & Kindig (2006), communities are not illiterate, and to a degree they can attain, understand and process simple information provided in order to make proper decisions. Austin et al. (2005) concur with the latter, since UDDTs require a higher level of commitment from users than do other forms of dry sanitation.

## OBJECTIVE

The objective of this study was to explore the acceptance, use and maintenance of the UDDT.

## METHODS

The study was undertaken using both quantitative and qualitative methods, which allowed a process of triangulation, as Non-maintainer This is the label given to households with the UDDT in a bad condition, i.e., it has broken items, and the broken items are either not repaired or repaired using inappropriate material questionnaires, focus group discussions (FGD) and in-depth Marshall&Rossman (1999) stated that triangulation is essential to check and establish the reliability and validity of the study

Initially, a questionnaire was used, and later FGD and in-depth interviews were held. The study was undertaken in three periurban areas of EM, namely, Zwelibomvu (west), Lower Maphephetheni (north) and Hlanzeni (south). These areas were selected as representing different geographical areas of EM where the UDDTs had been installed. In each of the three study areas, 40 households were randomly selected from an aerial map using the metro number which is allocated by eThekwini Water and Sanitation (EWS) to all households with a water or sanitation service. At each household a short questionnaire including a checklist was administered on the first visit to ascertain if the household was: (1) maintaining the toilet properly (maintainer), (2) not maintaining the toilet properly (non-maintainer) or (3) not using the toilet (non-user) (Table 1). This information helped in forming the subsequent focus groups, which were homogenous in nature (either maintainer, non-maintainer or non-user group), and this was done to encourage openness and transparency. Each of the 120 households visited was represented by one adult family member in the focus groups. In total, 121 people participated because one house-hold at Zwelibomvu was represented by two family members.

**Table 1:** Categories used for grouping participants (n=121) in the four discussions held in each of the three study areas in eThekwini

Category	Description
Maintainer	*This is the label that was given to households with the UDDT in a good condition*, i.e., all items intact, e.g., door, vent pipe, etc. in place. The broken items are repaired using appropriate materials
Non-maintainer	*This is the label given to households with the UDDT in a bad condition*, i.e., it has broken items, and the broken items are either not repaired or repaired using inappropriate material
Non-user	Households that have a UDDT but choose not to use it

In each study area, four FGD were held consisting of eight to 11 individuals in each group. Purposive sampling was used in selecting the key informants for the in-depth interviews, as they were selected according to their involvement in the UDDT project, the position they hold in their community and their knowledge about the UDDT. The key informants who introduced the UDDTs to households were ward committee members, ward councillors and previous local facilitators. In total, 25 people participated. Short, open-ended interview schedules were developed for these in-depth interviews and FGD to facilitate the discussions. Probing was used to elaborate and clarify the matters being discussed. All interviews and FGD were tape recorded, transcribed word for word in Zulu and translated into English while preserving the meaning by the researcher, who has experience in this area. The qualitative data were analysed manually through the process of content analysis, data were verified, categorisation of data was done, the categories were coded, and contrasts and similarities were identified and then the meaning was further explored. The study was approved by the University of KwaZulu-Natal (UKZN) Biomedical Research and Ethics Committee (BREC), ref: BE07/13

## RESULTS

The results are presented in two broad sections. The first section contains findings from the brief questionnaire that was mainly looking at the condition of the UDDT. The second section comprises findings from the FGDs and in-depth interviews, and is divided into acceptance, use, maintenance and education.

### Condition of the UDDT

The quantitative findings revealed that of the 97% of house-holds that were using the toilet, 80% were not maintaining the UDDT properly and only 17% maintained it properly. Households where the UDDT was maintained properly had more children.

**Figure 1:**
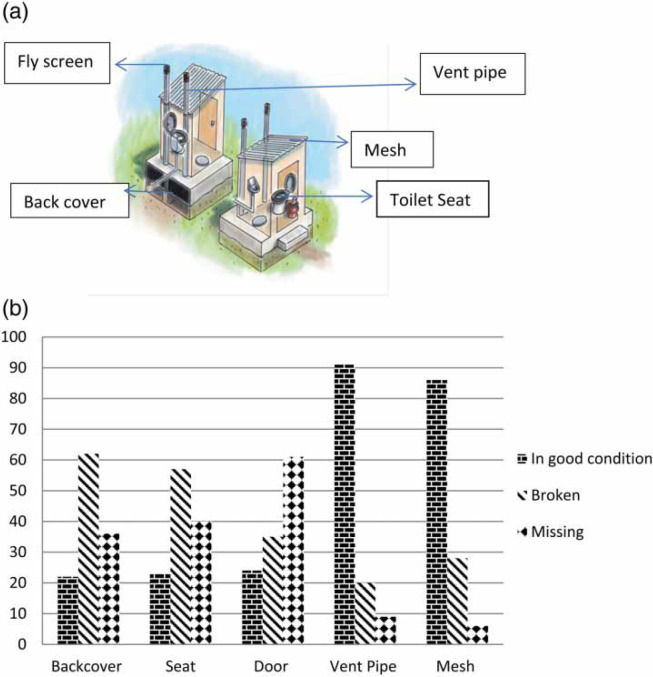
(a) UDDT showing different constituent items and (b) condition of UDDT constituent items (n=120).

Most of the UDDT items that were broken were the out-side door, back cover at the vault and the toilet seat (Figure 1(a) and 1(b)). The mesh and the vent pipe were items that were intact in most cases.

### Qualitative approach

#### Focus groups and in-depth interviews

The data presented in this section were gathered from 146 participants, of whom 110 were female; their age varied between 21 and 65 years. For many households, the reason for the choice of the person to represent them in the focus group was that their nominee had no commitments on that particular weekend

#### Acceptance of UDDT

Although the participants generally did not accept the UDDT (independent of the condition of the toilet), the younger participants were more accepting and they showed greater awareness as to why it was installed compared to the older participants, who often compared the UDDT to the VIP (ventilated improved pit latrine).

*I do not have a problem with using the UDDT; I have used it ever since I can remember*. (Female interviewee)

*I am okay about using the UDDT because I understand why they installed it*. (Male focus group member)

More than 95% of the participants reported that they do not regard the UDDT as a permanent asset and they all aspired to have a flush toilet, which is associated with being a first-class citizen.

*I’m waiting for my flush toilet, there is no way that this toilet is permanent…I will not accept that*. (Female focus group member).

The majority of the participants shared the view that the UDDT was the toilet for the poor.

*Wealthy people convert these toilets to flush or septic tank, so only us who cannot afford are still using them as we were given*. (Male interviewee)

**Figure 2:**
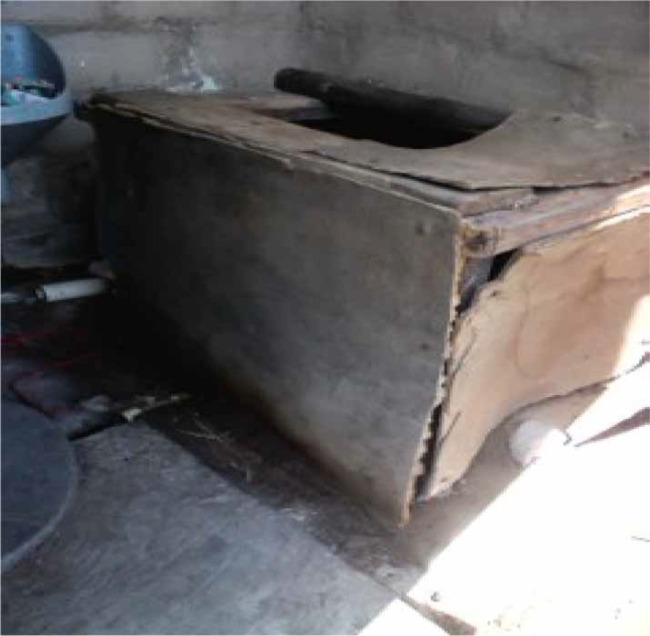
| UDDT seat repaired inappropriately.

The majority of the participants reported that they do not identify with the UDDT benefits, such as using urine as a fertiliser, or that the UDDT is cost-effective compared to the VIP toilet. Many did not concur as they felt that UDDTs are costly to maintain due to easily breakable items like doors and seats (Figure 2).

*Pity is that many of us do not have gardens anymore and we are not even interested so I do not think this (UDDT) works for people*. (Male who was a previous facilitator)

*The door broke a few months after the toilets were installed…the seats are unstable because they are weak. We have people who are big physically*. (Female interviewee)

The participants felt that the UDDT was not sensitive about their comfort since one has to be mindful all the time if your urine or faecal matter is going to the right place.

*It is too technical, having to make sure that the urine goes to which hole, it takes away the comfort and peace that one should get when using a toilet*. (Male ward councillor)

Additionally, participants reported that UDDTs are dark inside and this is the reason why most children do not close the door.

*A toilet should be a place where I can relax and even read*. (Female focus group participant)

A large proportion of people reported that they first heard about the toilets only a few days before the installation.

*If only we were informed of this toilet before they installed them, maybe we would have asked them to give us some-thing different*. (Female interviewee)

#### Use of the UDDT

Despite the low level of acceptance, the UDDT was used by 97% of the participants, but many reported that it was not by choice.

*We have no alternatives that is the reason we use this (UDDT)*. (Female interviewee)

The fear of allowing children between two years and five years to use the UDDT toilet was one of the highly discussed issues. The majority of the participants reported that they discouraged their children from using the UDDT and they practise OD instead. The main reason was the fear that they might fall, injure themselves or die.

*So to be safe, I would rather they did not use the toilet until they are old enough…at least be 6 years old*. (Female focus group member)

*I tell my grandchildren to use the open space by our house to defecate because the hole of the toilet seat is big, it’s risky*. (Female previous facilitator)

#### Maintenance of the UDDT

Among those who were users, 80% were not maintaining the UDDT properly and only 17% were maintaining it properly.

The maintenance was one of the main reasons many participants dislike the UDDT and why others choose not to use it. Emptying the UDDT caused tensions in many households because it is a job that many prefer not to do. The findings reveal that the UDDTs are mainly cleaned by females in the household, and this includes the emptying of the toilet. A small proportion of respondents reported that the task to empty the toilet is done by older females, because being in contact with faecal matter will bring bad luck to younger females.

*They might end up not getting married if they do that dirty job*. (Female focus group member)

*Females end up cleaning and emptying the toilet because keeping the house clean is our responsibility*. (Female focus group member)

*We chose not to use the toilet because no one was pre-pared to empty it, so now we use it as the store room*. (Male focus group member)

The participants reported that the toilets fill up quickly because the vault is shallow.

*If they can make vaults bigger so at least I empty the toilet once in a year…now I have to empty twice a year*. (Male interviewee)

The maintainer group reported that they worked as a team. They took turns to clean and empty the UDDT. Whereas in the non-maintainer groups, the chore to clean and empty the toilet was one person’s responsibility.

*It’s better to clean it knowing that next week it will be someone else…so when it’s your turn you want to make sure that you do not let other people down as well*. (Female focus group member)

*I get tired of cleaning it so at times I ignore it*. (Female interviewee member)

#### Lack of UDDT education

A large proportion of the participants reported that they were not direct recipients of the UDDT education, in some cases other family members had received the information, and in other cases they had bought the house after the UDDT was installed. Subsequently, many participants blamed lack of education on how they use and maintain the toilet. However, what was observed from the information gathered was that in the maintainer group the persons who received the UDDT information were younger, compared to the non-maintainer group.

*We never received education. The people came once to our house telling us the UDDT will be installed tomorrow*. (Male focus group member)

*I bought the house with this toilet and the previous owners never explained to me, so we had to figure it out ourselves*. (Female focus group member)

## DISCUSSION

### Acceptance

In this study, we explored the views and perceptions of the UDDT users in relation to acceptance, use and maintenance. The data show no direct relationship between the maintenance and the level of acceptance, because even those maintaining the toilet adequately still aspired to have their toilet changed from the UDDT to a flush toilet. The older generation preferred the VIP toilet because they are accustomed to it, it requires less responsibility from the user and mostly the user does not have to empty it. The older generation also appreciated the VIP because the vault is deeper, the waste is far from the user and it takes a long time to fill up. The younger participants also often mentioned the geographic and economic impracticality of providing flush toilets to rural communities, and this understanding contributed to their acceptance.

### Non-acceptance

The UDDT proved difficult to accept because it does not meet the user’s perceptions regarding a ‘first class citizen’s standard of living and level of convenience’. Eales (2008) reported that poor South Africans (after the service discrimination experienced during apartheid) aspire to the same infrastructural services offered to the white population, which in this case is in-house flush toilets and piped water. Households that could afford it were changing their UDDTs to flush, and this included their community leaders. This emphasised the perception that a flush toilet is for rich people and that the UDDT is a symbol of poverty. Roma *et al*. (2013) found that 8.4% of respondents had converted their UDDTs to flush toilets. As discussed above, users reported many challenges with the UDDT that made it difficult for them to accept it. One of the benefits of the UDDT is the recycling of waste to be used as a fertiliser. However, in this study, users reported that this is not an incentive because very few people have gardens or are interested in gardening. Education and community participation will help EM understand what people’s aspirations are and what they regard as benefits. Furthermore, repairing the UDDT makes it a costly asset that most cannot afford. Murray *et al*. (2011) suggests that experts need to first establish which benefits will appeal to the community, and then the sanitation system design can be introduced based on this demand. A lack of consultation is one of the issues that was emphasised. EM reports that the community leadership was consulted and informed, but users’ reports disagreed; they explained that the information was not properly and timely provided. Consultation helps to provide accurate information, reduce negative perceptions and communicate expectations, concerns, fears and preferences of all those involved and this appeared to be lacking, thus more community participation is therefore necessary.

As reported by Matsebe (2011), emptying is one of the reasons that the UDDT is so unpopular with its users. It is one of the reasons reported in this study that perpetuates the perception that it is for poor people. Participants felt that cleaning the UDDT was unsafe for their health and made them feel undermined, although the myth that being in contact with faecal matter brings bad luck especially to younger people was rarely reported. Inferior or incorrect design and construction of the UDDT was high-lighted as a barrier to acceptance. They felt that most of the community were big in body size and the limited space of the UDDT was inconvenient and that the toilet seat broke easily. EM thus needs to increase the toilet size and to work with local businesses in stocking UDDT material that is durable, at a cost that people can afford. For the EM to respond to the desires of the users, they will need to use (a) education and community participation, (b) to modify the UDDT by increasing the size and to allow more light inside the toilet and (c) to use an alternative technology as more progress is made in developing models of ecological sanitation.

### Use

The findings show that a large proportion of people are using the UDDT, and this indicates that they see a need and the benefits of using the toilet. However, children under five years were encouraged to practise OD because parents fear that they might fall into the toilet. The participants reported that monitoring the child while using the toilet is time-consuming, but at the same time, they cannot risk sending the child alone into the toilet. Children’s faeces are considered to be not as harmful as that of adults, thus not realising the health hazards this poses to the family and the community at large. Moreover, it is a lost opportunity to teach children at a young age how to use a toilet, so that they can grow up with the correct attitude and behaviour, since using a toilet is a learned behaviour. The 3% of the respondents who chose not to use the UDDT mostly objected to emptying the faecal matter, and this group continued using basic pit latrines.

### Maintenance

Poor maintenance was indicative of the low acceptance and the perception that the UDDT is a temporary or interim measure. Although the majority of the participants were using the UDDT it was not their preferred choice, and this encouraged neglect of the UDDT. The doors, back covers and seats were reported to be items that easily break, which made people feel that they were given cheap toilets that were not customised to their reality. Additionally, McConville & Rosemarin (2012) found that users complain about the space, in that it was small and dark inside the toilet which made using the toilet uncomfortable. Future designs of UDDTs need to be of larger size, and two layers of bricks may need to be replaced by a translucent plastic material so as to allow more light inside the toilet. More-over, the EM in future will need to introduce other types of ecological sanitation facilities in order to offer communities a choice. The reason the UDDT was not maintained properly was because broken items were not repaired due to a lack of money and skills. In most cases, this resulted in the UDDT not functioning properly. The maintainers complained about easy breakages of the UDDT, but they repaired the problem, whereas non-maintainers would repair inadequately (Figure 2). EM will need to ensure that suitable materials are used in the construction to reduce such problems.

The other maintenance issue was toilets that were full to capacity but the vaults were either not changed or emptied, and this is where the great difference occurred between maintainers and the non-maintainers.

The households where the UDDT was properly maintained had more children, and they played a vital role in the maintenance of the UDDT. The latter suggests that children need to be involved when the sanitation technology is introduced because they are important in the use and maintenance of the UDDT. Children above six years of age use the UDDT toilet; however, the participants complained that children fail in most cases to urinate and defecate in the correct hole. Therefore, toilet seats that are suitable for children under five need to be provided and installed by EM after they have been checked and passed by the South African Bureau of Standards (SABS) for safety, so proper toilet use is encouraged from an early age. This study also revealed that females, both young and old, are mainly responsible for cleaning, but that emptying is mostly done by the older women due to the belief that being in contact with the faecal matter brings bad luck. This could be the reason why young people were more accepting of the UDDT than the older participants, since they do not have to perform what most people considered the worst aspect about the UDD toilet: the emptying.

Participants reported that most of the women are at home during the day, while men, although unemployed, were not at home and this had an influence on the division of tasks in the household. Drangert (2004) says this norm is seen mainly in Africa and it was also observed in Mexico. Moreover, as con-firmed in this study, there were more females than males who participated and it was evident that most males did not participate in the cleaning of the toilet. Many studies look at the maintenance but they do not consider which methods work, as this study attempted to do. In exploring this issue, we found that in families where males and females work together as a team in looking after the toilet they manage to maintain it properly. Among non-maintainers, where the task of cleaning the UDDT devolved around one household member, this chore was undertaken with frustration and the toilet was not well maintained.

### Education

The study found that many participants reported that they did not receive health and hygiene education from EM at the time of the UDDT installation because the direct recipients either did not relay the information to other family members, or they provided very little information. This suggests that relying on one member of the family to pass the information to other members, as EM previously did, is unreliable and there is a need to target the education to all or most family members, so that they all get first-hand information, which increases the level of accountability. Moreover, some of the users were new household owners, who had bought the house with the UDDT, and the previous owners never explained how to use it. This reality requires education to be provided on a regular basis to bridge the gap created by migration. However, in this study, the maintainer group was more informed about the UDDT than other groups and this suggests that keeping users informed will contribute towards proper use and maintenance of the UDDT. Health and hygiene education is one tool that can be used to actively involve the community, from planning to the implementation of the sanitation project, and this will ensure that the needs of the community are catered for.

## CONCLUSION

This study has highlighted the lack of understanding about the use and benefits of the UDDT by many community members. Although the UDDT was used by 97% of respondents, there was a low level of acceptance and many aspired to have a flush toilet. Lack of education concerning the use of the UDDT was evident, as were problems with the quality of the UDDT materials. Community participation and provision of education about the use of the UDDT are important strategies in changing perceptions about ecological sanitation in eThekwini. Moreover, the current drought in South Africa is a reminder that waterborne sanitation is not a feasible option; the emphasis needs to be on community participation and education to counter perceptions of an inferior product, which will improve acceptance, use and maintenance of the UDDT.
